# Characterization of a novel yeast phase-specific antigen expressed during in vitro thermal phase transition of *Talaromyces marneffei*

**DOI:** 10.1038/s41598-020-78178-5

**Published:** 2020-12-03

**Authors:** Kritsada Pruksaphon, Mc Millan Nicol Ching, Joshua D. Nosanchuk, Anna Kaltsas, Kavi Ratanabanangkoon, Sittiruk Roytrakul, Luis R. Martinez, Sirida Youngchim

**Affiliations:** 1 Graduate Program in Microbiology, Faculty of Medicine, Chiang Mai University, Chiang Mai, Thailand; 2 Department of Tropical Medicine, Medical Microbiology and Pharmacology, John A. Burns School of Medicine, University of Hawaii at Mānoa, Honolulu, HI 96813 USA; 3 Cellular and Molecular Medicine, Johns Hopkins University School of Medicine, Baltimore, MD 21205 USA; 4 Division of Infectious Diseases, Department of Medicine, Albert Einstein College of Medicine, Bronx, NY 10461 USA; 5 Infectious Disease Service, Department of Medicine, Memorial Sloan Kettering Cancer Center, New York, USA; 6 Department of Medicine, Joan and Sanford Weill Cornell Medical College, Cornell University, New York, NY 10065 USA; 7 Department of Microbiology, Faculty of Science, Mahidol University, Bangkok, Thailand; 8 National Center for Genetic Engineering and Biotechnology (BIOTEC), National Science and Technology Development Agency, Pathumthani, Thailand; 9 Department of Oral Biology, College of Dentistry, University of Florida, Gainesville, FL 32610 USA; 10 Department of Microbiology, Faculty of Medicine, Chiang Mai University, Chiang Mai, 50200 Thailand

**Keywords:** Microbiology, Fungi, Fungal pathogenesis

## Abstract

*Talaromyces marneffei* is a dimorphic fungus that has emerged as an opportunistic pathogen particularly in individuals with HIV/AIDS. Since its dimorphism has been associated with its virulence, the transition from mold to yeast-like cells might be important for fungal pathogenesis, including its survival inside of phagocytic host cells. We investigated the expression of yeast antigen of *T.* *marneffei* using a yeast-specific monoclonal antibody (MAb) 4D1 during phase transition. We found that MAb 4D1 recognizes and binds to antigenic epitopes on the surface of yeast cells. Antibody to antigenic determinant binding was associated with time of exposure, mold to yeast conversion, and mammalian temperature. We also demonstrated that MAb 4D1 binds to and recognizes conidia to yeast cells’ transition inside of a human monocyte-like THP-1 cells line. Our studies are important because we demonstrated that MAb 4D1 can be used as a tool to study *T.* *marneffei* virulence, furthering the understanding of the therapeutic potential of passive immunity in this fungal pathogenesis.

## Introduction

The thermally dimorphic fungus *Talaromyces* (*Penicillium*) *marneffei* causes a life-threatening systemic mycosis in immunocompromised individuals living in or traveling to Southeast Asia, mainland of China, and the Indian sub-continents. *Talaromyces* *marneffei* is a distinctive species in the genus. It is the only one species capable of thermal dimorphism. The dimorphism exhibited by *T.* *marneffei* is controlled by the cultured temperature of the fungus^1^. At room temperature (25–28 °C), the fungus undergoes asexual development to form filamentous growth with mycelial cultures common to the genus *Talaromyces*. In contrast, at higher temperatures the fungus changes from a filamentous mold to a yeast form, dividing via fission at 37 °C or during infection, which appears to be requisite for pathogenicity.

The morphologic switching from the mold to the yeast form of dimorphic fungi is needed for virulence^2^. When dimorphic fungi change from an environmental mold morphotype to the pathogenic yeast morphotype, this results in an alteration not only in cell shape, but also in an expression of yeast-phase specific-protein involved in the virulence of the dimorphic fungi. Therefore, the transition to yeast or yeast-like growth results in altered cell wall composition as well as in the production of proteins used to evade immune defenses or toxins to alter host behavior. Growth as a yeast coupled with thermotolerance allows for replication within phagocytic cells, which assists systemic dissemination^3^. After the deletion of a dimorphic controlling gene, the lost ability of a fungus to undergo switching has been shown to limit its virulence in various dimorphic fungi including *T.* *marneffei*^4,5^.

Currently, there are relatively few studies investigating the cellular protein differences between the mycelial and yeast phases of *T.* *marneffei* or the intracellular events that correlate with transformation of the fungus. Proteomic profile studies in the *T.* *marneffei* strain PM1 have identified 12 proteins which are differentially expressed in *T.* *marneffei* between mycelial and yeast phases. Eight of these proteins are highly expressed in the yeast phase of *T.* *marneffei*, and several of these proteins have sequences homologous to various housekeeping metabolic enzymes such as malate dehydrogenase, fructose bisphosphate aldolase, dihydrolipoamide dehydrogenase, and heat shock protein 60 (HSP60)^6^.

Yeast phase specific monoclonal antibody 4D1 (MAb 4D1) binds to the *T.* *marneffei* cytoplasmic yeast antigen (TM CYA) and reacts against a 50–150 kDa of N-linked glycoprotein with high molecular mass. However, it failed to react with the mycelial phase cytoplasmic antigen of *T.* *marneffei* (TM CMA)^7−10^. It is possible that the antigenic glycoprotein recognized by MAb 4D1 is only expressed in the *T.* *marneffei* yeast phase. Due to the difficulties and inconsistencies of the tool for investigating the basic biology of phase transition in *T.* *marneffei*, in vitro phase transition models (especially in host macrophages) remain essential for studying the virulence factors and pathogenicity of this dimorphic fungus^11^. Therefore, the goal of this study is to characterize the expression profiles between mold and yeast phase transition and the biochemical nature of *T.* *marneffei* antigenic glycoproteins recognized by MAb 4D1. Furthermore, our study demonstrates that the yeast phase specific antigenic glycoprotein recognized by MAb 4D1 might be a novel candidate marker for tracking cellular events during the in vitro thermally induced phase transition.

## Results

### The specificity of MAb 4D1 to the yeast phase antigen of *T. marneffei* was shown by indirect ELISA and fluorescent microscopy

**Figure 1:**
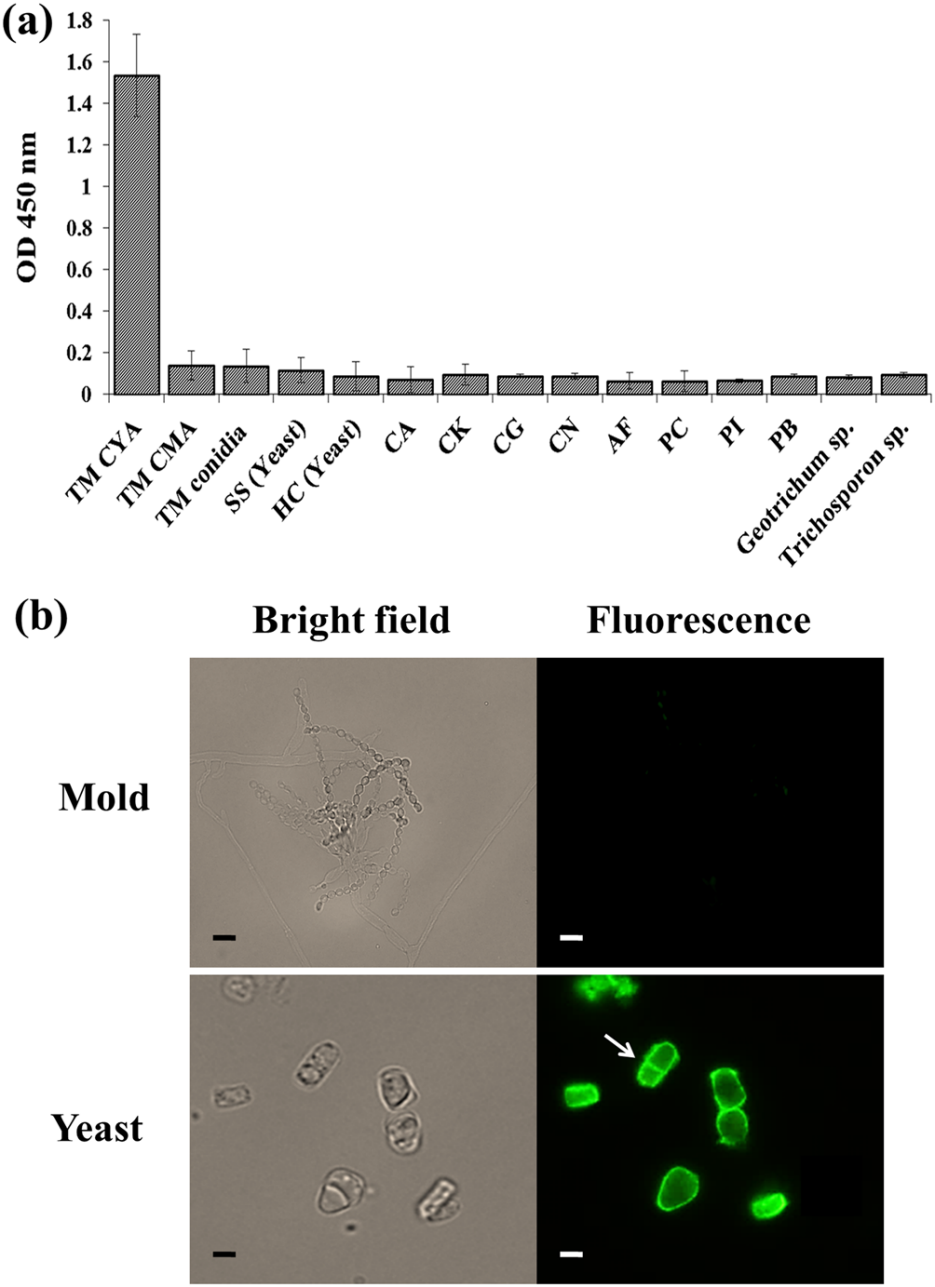
The specific immunoreactivity of MAb 4D1 against yeast phase antigen of *T. marneffei*. (**a**) Indirect ELISA. (**b**) Corresponding bright fields and indirect IFA showing immunoreactivity of MAb 4D1 against *T. marneffei* mold and yeast culture, the arrow indicated the fission yeast cell. TM; *Talaromyces marneffei*, SS; *Sporothrix schenckii*, HC; *Histoplasma capsulatum*, CA; *Candida albicans,* CK; *Candida krusei*, CG; *Candida glabrata*, CN; *Cryptococcus neoformans*, AF; *Aspergillus fumigatus*, PC; *Penicillium citrinum*, PI; *Pythium insidiosum*, PB; *Pseudallescheria boydii*.

MAb 4D1 was shown by indirect ELISA to react specifically to TM CYA without cross reactivity to either *T.* *marneffei* cytoplasmic mold antigen or cytoplasmic conidial antigen. In addition, no immunoreactivity against a panel of dimorphic and common fungal antigens (e.g. *Histoplasma* *capsulatum*, *Candida* *albicans*, *Cryptococcus* *neoformans* and *Aspergillus* *fumigatus)* was observed (Fig. 1a). Fluorescent microscopy demonstrated that MAb 4D1 only recognizes the cell wall of yeast cells. In contrast, the mold form of *T.* *marneffei* failed to react with MAb 4D1 (Fig. 1b). Therefore, our findings indicate that MAb 4D1 is highly specific against only the yeast phase antigen of *T.* *marneffei*.

### Immunoreactivity patterns and carbohydrate components of the antigenic proteins recognized by MAb 4D1 and *Galanthus nivalis* agglutinin (GNA)

**Figure 2:**
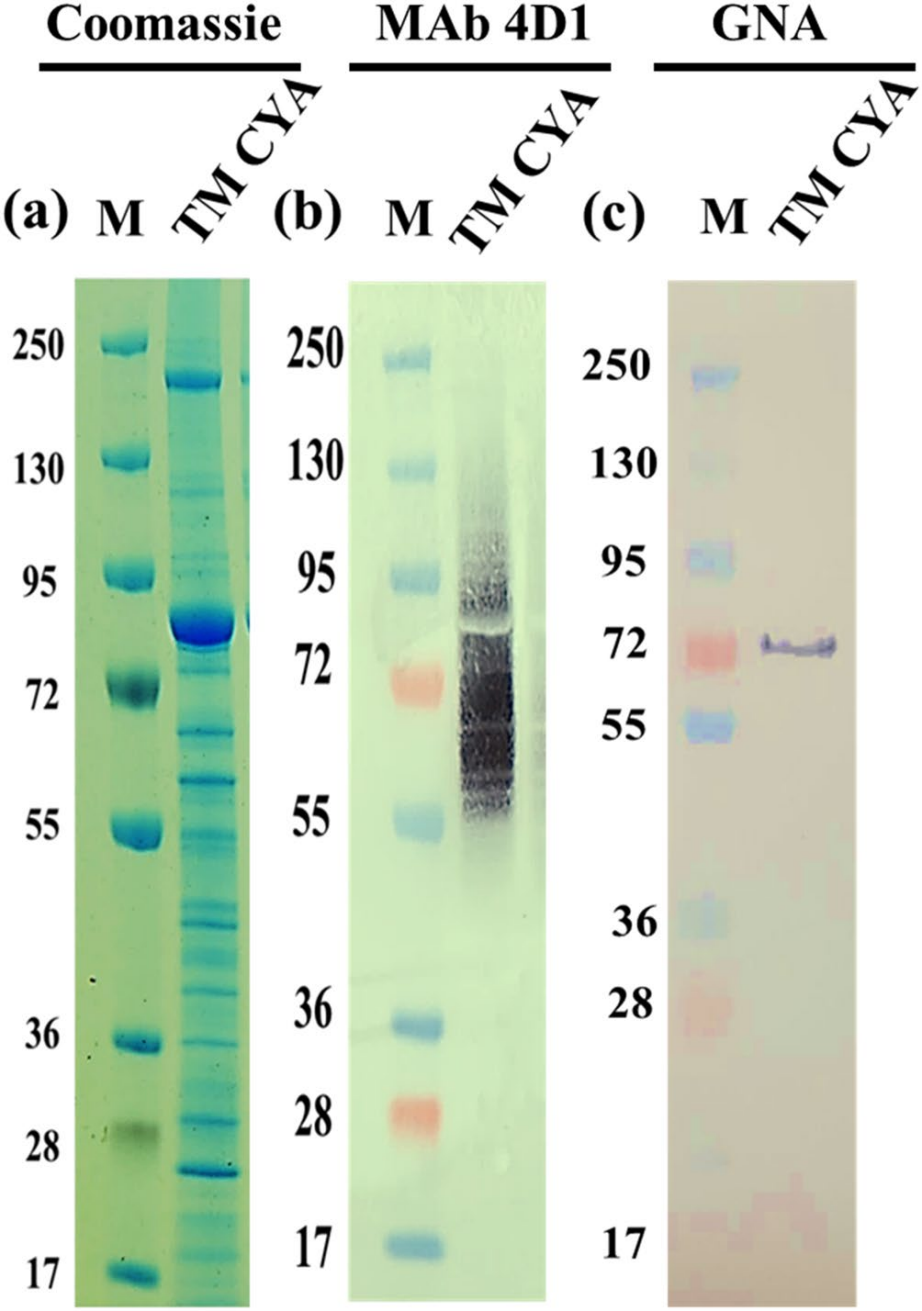
Biochemical characteristics of *T. marneffei* cytoplasmic yeast antigen or TM CYA. (**a**) SDS-PAGE showing the antigenic profile of the TM CYA stained with Coomassie blue. (**b**) Native TM CYA recognized by MAb 4D1 with a molecular weight ranging 50–150 kDa with the diffuse binding characteristic of “broad high molecular mass smear” (**c**) TM CYA recognized by HRP-GNA with the molecular weight approximately 72 kDa. (M: Pre-staining molecular weight marker; kDa).

The cytoplasmic protein components of TM CYA were separated on SDS-PAGE and stained with Coomassie blue. Over 20 protein bands with molecular weights (MW) ranging from 17 to 250 kDa were observed. The most abundant bands showed MW between 72 and 95 kDa (Fig. 2a). By immunoblot, the epitopes recognized by MAb 4D1 appeared to distribute among multiple undefined protein bands with broad high molecular mass, between 50 and 150 kDa (Fig. 2b) similar to data previously described^7–10^. These results indicate that the target epitope of MAb 4D1 is shared by various forms of the glycoprotein according to previously described findings^12^. The GNA binding studies demonstrated that the majority of carbohydrate components in TM CYA consisted predominantly of mannose residues. Only one band with molecular weight approximately 72 kDa was recognized by HRP-GNA (Fig. 2c). GNA is highly specific for α 1, 3 linked non-reducing terminal mannose residues in either N- (asparagine) or O- (serine, threonine, and tyrosine) linked glycosylation^13,14^. After that, we carried out the LC–MS analysis of the 72 kDa antigen of TM CYA recognized by HRP-GNA with Mascot software and the NCBI database. These 72 kDa antigens showed a strong homology with the *katG* catalase-peroxidase enzyme (KATG_TALMA) of *T.* *marneffei* (Table 1).These results suggest that MAb 4D1 recognizes multiple epitopes in the mannoprotein of TM CYA.

**Table 1:** Proteomic analysis of the 72 kDa antigen of TM CYA recognized by *Galanthus nivalis* agglutinin (GNA).

Accession number	Gene	Protein name	Organism	Score	Protein sequence coverage	Monoisoto-pic mass (KDa)	Calculated pI
Q8NJN2 (KATG_TALMA)	*katG*	Catalase-peroxidase	*T. marneffei* (*P.marneffei*)	70	6%	82.38	6.35

### Effects of peptide-N-glycosidase F (PNGase F) and proteinase K treated TM CYA altered antigenic recognition by MAb 4D1

**Figure 3:**
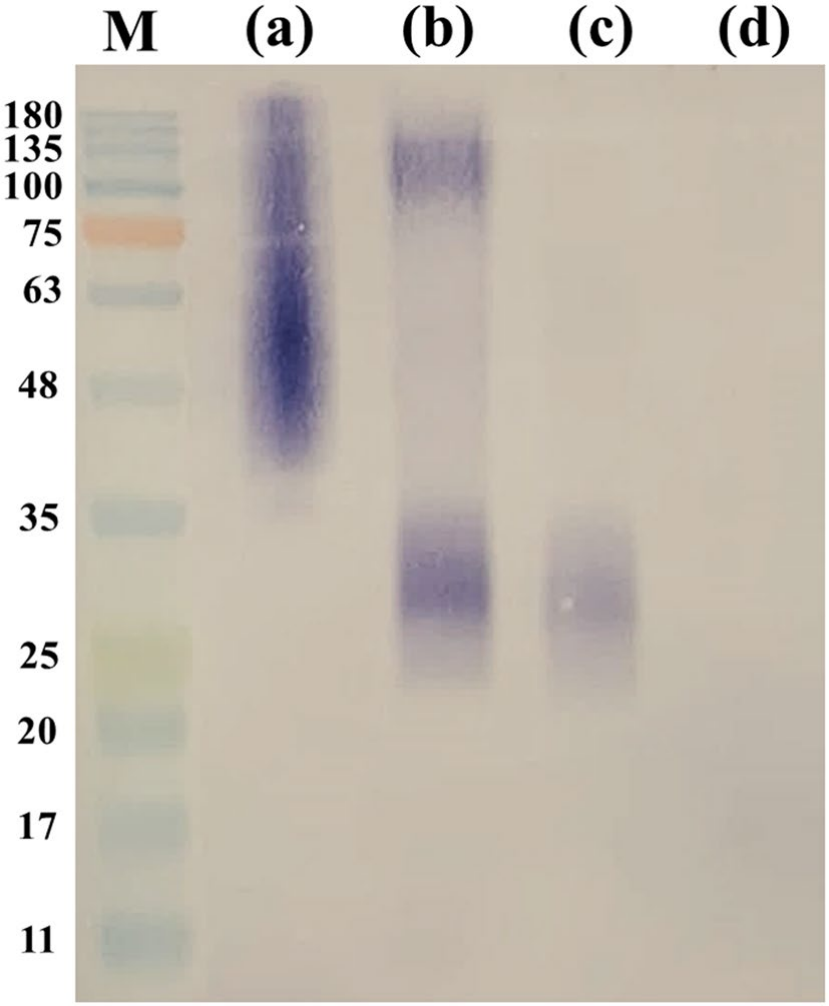
The effect of N-linked deglycosylation of TM CYA on antigenic recognition by MAb 4D1. (**a**) Native recognition patterns of TM CYA by MAb 4D1. (**b**) Recognition patterns of TM CYA by MAb 4D1 after digested with PNGase F at 90 min. (**c**) Complete recognition patterns of TM CYA by MAb 4D1 after digestion with PNGase F at 180 min. (**d**) *T. marneffei* cytoplasmic mycelial antigen or TM CMA used as a negative control.

**Figure 4:**
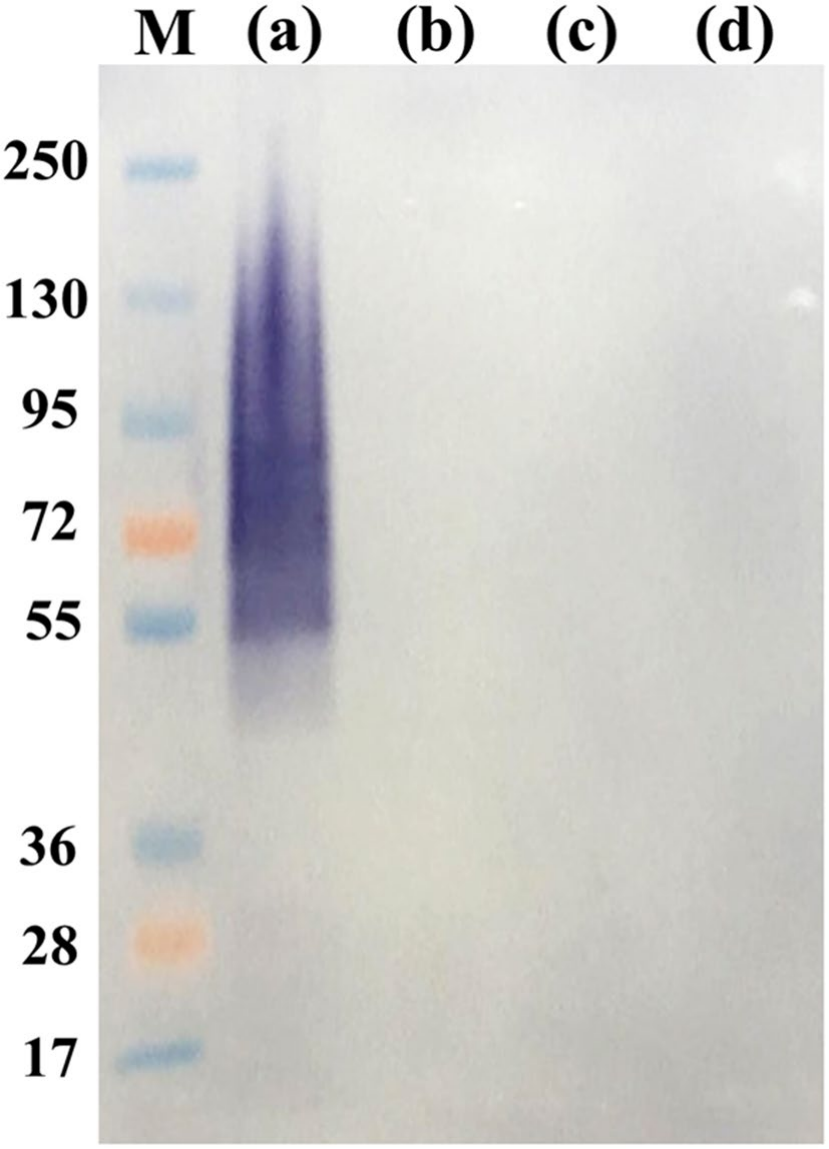
The effect of proteinase K treated TM CYA showing altered antigenic recognition by MAb 4D1. (**a**) Native recognition patterns of TM CYA by MAb 4D1. (**b**) Recognition patterns of TM CYA by MAb 4D1 after digestion with proteinase K at 30 min. (**c**) Recognition patterns of TM CYA by MAb 4D1 after digestion with proteinase K at 60 min. (**d**) Proteinase K treated *T. marneffei* cytoplasmic mycelial antigen or TM CMA used as a negative control.

Following treatment of TM CYA with PNGase F at varying digestion times, the different characteristics of the native glycosylated forms and the deglygosylated TM CYA were demonstrated (Fig. 3). In the native glycosylated form, MAb 4D1 stained the proteins as a broad high molecular mass smear (Fig. 3a). In contrast, after deglycosylation with PNGase F for 90 min, the band was different (Fig. 3b), demonstrating that the glycosylations in TM CYA are heterogeneous. After 180 min of digestion, the completely deglycosylated TM CYA showed relatively diffuse bands with molecular masses of approximately 35–25 kDa (Fig. 3c). However, further digestion time (overnight incubation) did not result in considerable changes in the MAb 4D1 stained protein profiles (data not shown). This result revealed that enzymatic removal of N-linked glycosidic bond from TM CYA, by treatment with recombinant PNGase F resulted in an altered recognition pattern of TM CYA by MAb 4D1. Consequently, the absence of MAb 4D1 immunoreactivity against TM CYA in the immunoblotting following treatment of TM CYA with proteinase K at 30 and 60 min, this implied that the target epitope of MAb 4D1 associated with peptide (Fig. 4).

### Recognition of antigenic proteins by MAb 4D1 during the thermally induced phase transition of *T. marneffei*

**Figure 5:**
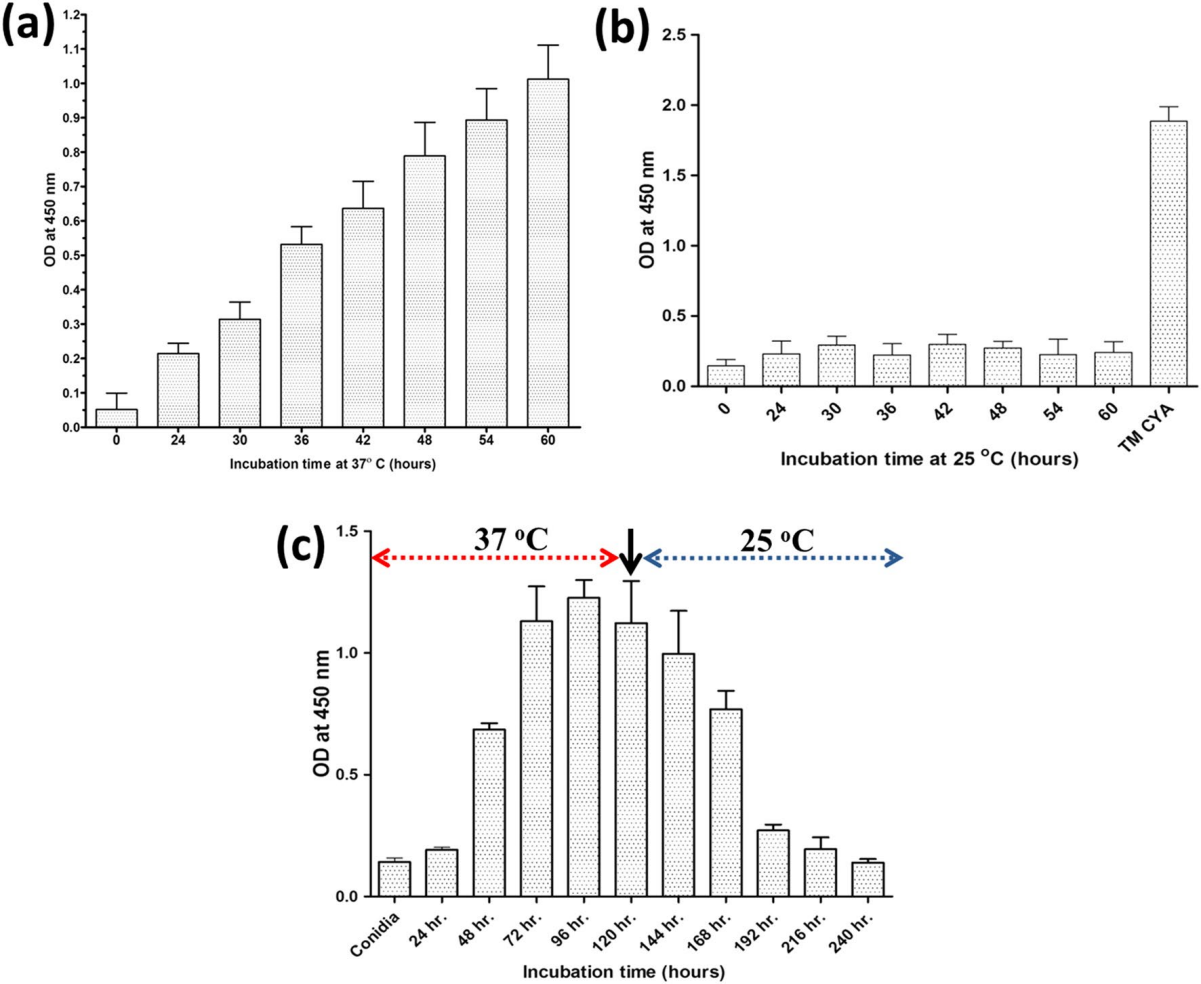
Immunoreactivity of MAb 4D1 against cytoplasmic antigen from *T. marneffei* subjected to BHI culture temperature shift at (**a**) 37 °C, and (**b**) 25 °C after 0, 24, 30, 36, 42, 48, 54, and 60 h, respectively. (**c**) Culture temperature shift from 25 to 37 °C, and from 37 to 25 °C, with the arrow indicating shifting time point.

Indirect ELISA analysis confirmed that MAb 4D1 demonstrated reactivity against TM CYA. *T.* *marneffei* was cultured in brain heart infusion (BHI) broth medium at 37 °C for 24–60 h and each sample was collected every 6 h. We observed that the increase in immunoreactivity against TM CYA was directly proportional to the time of incubation (Fig. 5a). In contrast, minimal immunoreactivity against the cytoplasmic mycelial antigens recovered from *T.* *marneffei* was observed during incubation at 25 °C (Fig. 5b). We investigated whether conidia shift to yeast and yeast shift to mycelia alters MAb 4D1 immunoreactivity. At 37 °C, we found an increase in immunoreactivity of MAb 4D1 to cytoplasmic antigen recovered from *T.* *marneffei* cells after 48 h incubation and this gradually increased until 96 h. However, a decreased in immunoreactivity was observed after 24 h after the temperature was shifted from 37 to 25 °C, and the degree of immunoreactivity decreased gradually with the prolonged culture at 25 °C (Fig. 5c). These data demonstrate that MAb 4D1 binding is specific to the yeast phase of *T.* *marneffei* and is modulated by mammalian body temperature and the transitioning speed from mold to yeast phase.

### Recognition of yeast phase specific antigens by MAb 4D1 during *T. marneffei* conidia transition in vitro (1% proteose peptone) as studied by flow cytometry

**Figure 6:**
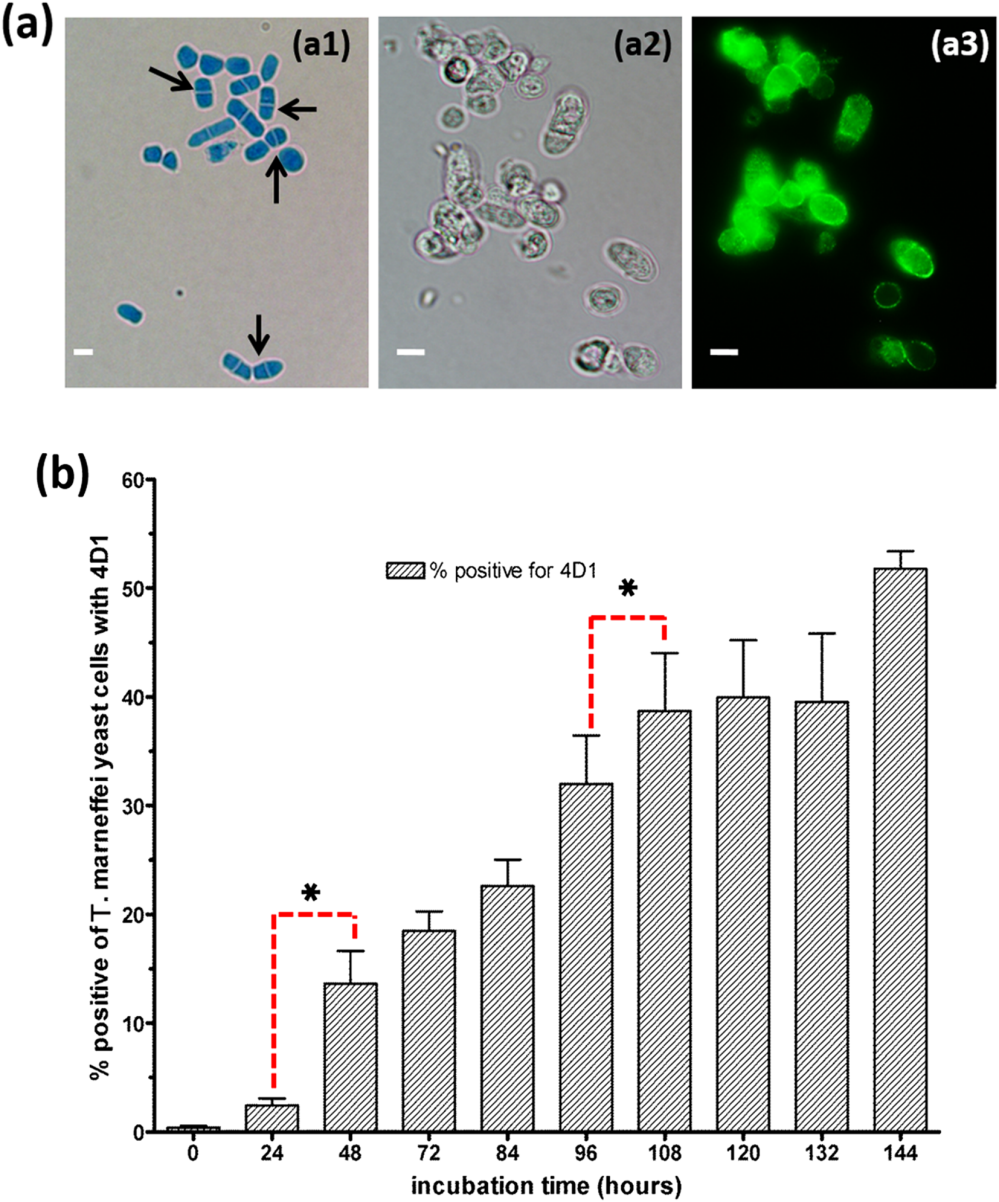
The morphological transition of *T. marneffei* yeast cell in 1% proteose peptone. (**a1**) *T. marneffei* yeast cell after 48 h of conidial inoculation in 1% proteose peptone stained with lactophenol cotton blue, the arrow indicating the fission yeast cell.(**a2**,**a3**) Corresponding bright fields and indirect IFA showing the immunoreactivity of MAb 4D1 against *T. marneffei* yeast cells cultured in 1% proteose peptone. (**b**) The percentages of MAb 4D1 surface labeled fluorescent positive yeast cells**,** each bar represents the mean ± SD of 3 sets of independent determinations. An asterisk (*) indicates a statistically significant difference (*p* < *0.05*) between transit time of 24 to 48 h and 96 to 108 h.

The yeast antigen expressed from *T.* *marneffei* cells undergoing thermal phase transition was analyzed by indirect immunofluorescence and quantified by flow cytometry. In 1% proteose peptone, *T.* *marneffei* conidia readily converted to fission yeasts with a transverse septum, which is the *T.* *marneffei* morphotype seen in clinical specimens^15,16^ (Fig. 6a1). *T.* *marneffei* conidia were cultured in 1% proteose peptone at 37 °C and the cells were harvested at time intervals of 12 or 24 h, between 24 to 144 h. The percentages of MAb 4D1 surface labeled fluorescent yeast cells were gradually increased after 48 h of incubation time. The maximum fluorescent positive yeast cells were quantified in 108–144 h after inoculation into 1% proteose peptone (Fig. 6b). This observation corresponded with the indirect ELISA results described above.

Taken together, these results demonstrate that the in vitro artificial cultivation medium, BHI and 1% proteose peptone could initially induce yeast phase specific antigen expression within 36–48 h. Moreover, these results confirm that *T.* *marneffei* yeast phase specific antigen remains expressed in cell cytoplasm and translocated to cell surface of *T.* *marneffei* yeast cells (Fig. 6a2,a3).

### Flow cytometric determination of yeast cells specific antigens using MAb 4D1 during *T. marneffei* phagocytosis by THP-1 cells

**Figure 7:**
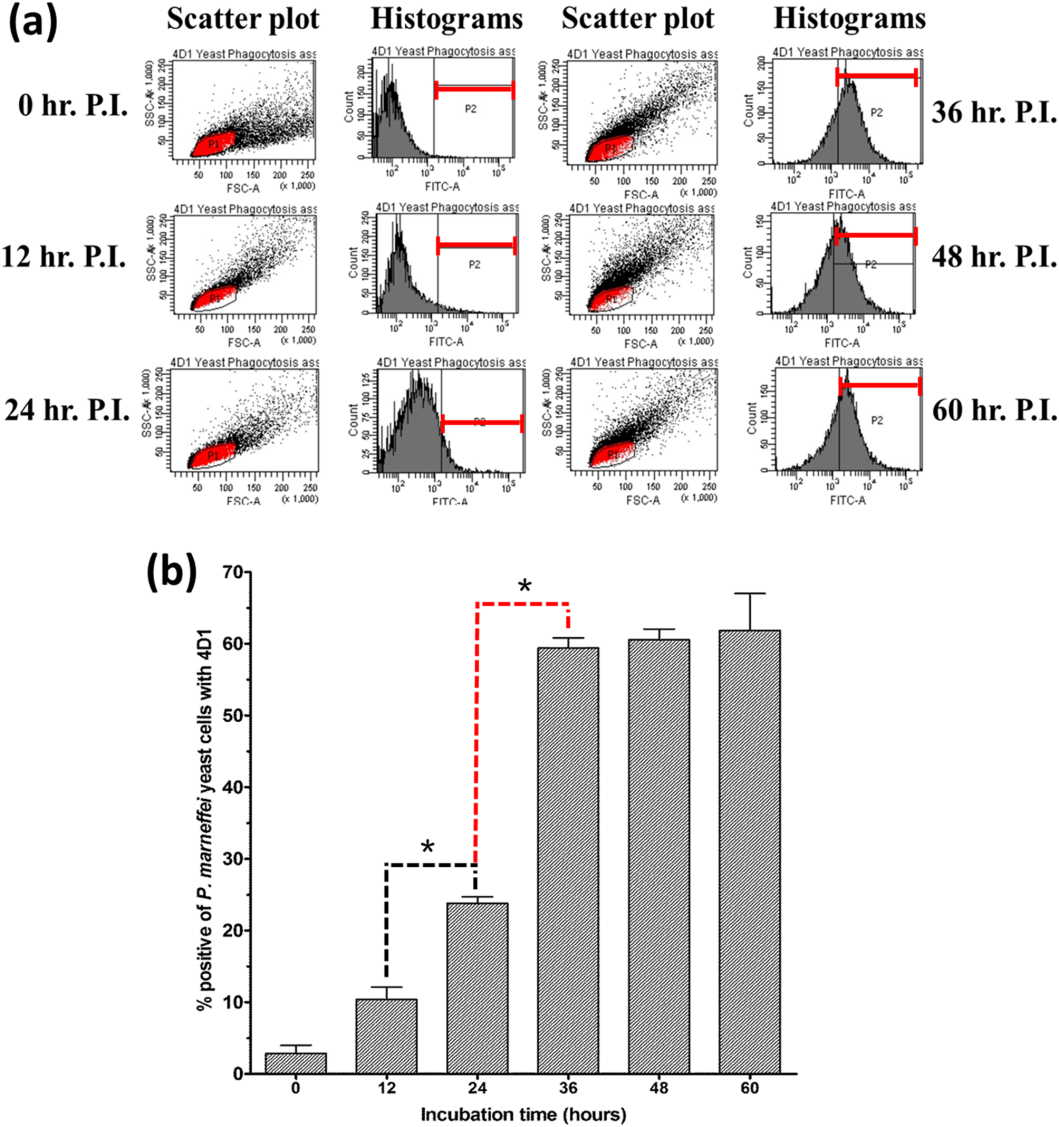
Reactivity of anti-yeast specific MAb 4D1 against *T. marneffei* yeast cells’ uptake by THP-1 cells at different time points as studied by flow cytometer. (**a**) The scatter plot panel illustrates the distribution patterns of the fungal particles analyzed according to forward scatter and side scatter of each P.I. time point. Whereas, the histograms demonstrate the relative fluorescence intensity shift of MAb 4D1 positive yeast cell of each P.I. time point. (**b**) The averages % of positive yeast cells ± SD of 3 sets of independent experiments was shown for each time point. An asterisk (*) indicates a statistically significant difference (*p* < *0.05*) between transit times at initial expression between 12 to 24 h and maximal expression of 24 to 36 h (P.I. post of internalization).

The ability of *T.* *marneffei* conidia to transform into yeast cells at 37 °C during internalization by THP-1 human macrophage was investigated. This study was carried out at different time points of 0, 12, 24, 36, 48 and 60 h. After the THP-1 membrane was disrupted with 1% triton X-100, the released *T.* *marneffei* cells were stained with MAb 4D1 and fluorochrome antibody conjugate as described in the methods. Figure 7a shows the scatter plot and histograms analyzed by flow cytometry. P1 channel in the scatter plot represents the yeast cell population that was selected to be analyzed by MAb 4D1 surface labeled positive yeast cells. In contrast, the P2 channel in the histogram represents the normalized fluorescent intensity of MAb 4D1 surface labeling positive.

The percentages of MAb 4D1 surface labeled fluorescent yeast cells were initially measured within 12 h of incubation time (10.28%) and then continuously increased after 24 h (23.8%) of incubation time. The maximum of fluorescent positive yeast cells were quantified within 36 h (61.85%) and remained at a similar level after 48 h (Fig. 7b). When compared to the results in artificial cultivation medium, this experiment demonstrated that the phase transitional ability of *T.* *marneffei* conidia in culture medium was converted to yeast cells at a slower rate than that in the host macrophage THP-1 environment.

### Recognition of *T. marneffei* yeast phase specific antigens by MAb 4D1 during conidial internalization by human THP-1 macrophage cells

**Figure 8:**
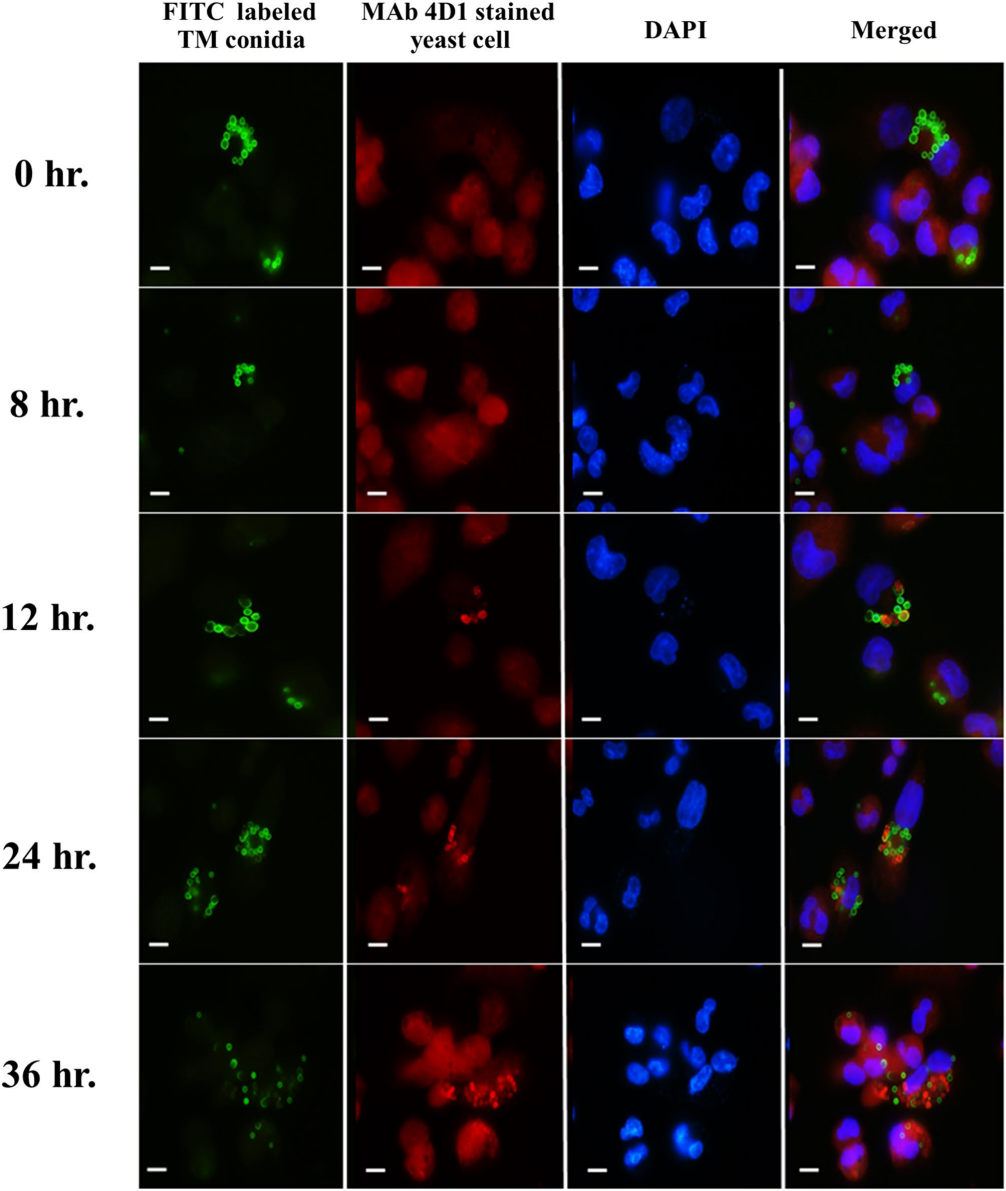
Internalization of *T. marneffei* conidia by THP-1 cells and intracellular germination to give rise to fission yeast cells. THP-1 cells were incubated with FITC-labeled *T. marneffei* conidia for 2 h, and then washed to remove unbound conidia. The THP-1 cells were further incubated for an additional time points 0, 8, 12, 24 and 36 h. At each time point, *T. marneffei* yeast cells were labeled with MAb 4D1 and Alexaflor 555 conjugated goat anti-mouse IgG antibody. From left to right: fluorescence image showing the green channel (FITC labeled conidia); fluorescence image of the red channel (MAb 4D1 positive yeast cells); THP-1 nuclei were stained with DAPI (blue); a merged channel showing the overlapping of triple images. Bars, 5 µm.

By tracking the intracellular phase transition of conidia to yeast inside the THP-1 human macrophage, it was shown that many FITC labeled *T.* *marneffei* conidia were rapidly internalized by THP-1 macrophages. These results demonstrate the ability of the fungus to survive before converting from conidial to yeast form, and then replicates inside the macrophages. (Supplementary Fig. S1). At less than 8 h after internalization, *T.* *marneffei* labeled FITC conidia were bright green of the mold phase. After 8 h, the conidia were swelling but the green color still covered the conidial wall. The red signal of yeast phase-specific antigen appeared after 12 h of macrophage internalization, and started to transit into yeast phase. The red signal was progressively observed and completely positive for MAb 4D1 after 36 h of macrophage internalization, indicating that the conidia were completely changed to yeast cells (Fig. 8). Furthermore, a similar phenomenon increasingly progressed at 48 h after conidial internalization (Fig. 8).

In addition, we demonstrated that the conidia were directly converted to fission yeast cells along with the expression of the yeast specific antigen 12 h after phagocytosis. These phenomena were clearly observed by overlapping signals between the green color of FITC labeled conidia and the red of MAb4D1 which gives the co-localized signal as a yellow at 12 h after internalization by macrophage (Supplementary Fig. S2). This observation is consistent with previous studies, wherein Andrianopoulos and colleagues suggested that the conversion of conidia to unicellular yeast morphogenesis program might be triggered by acidic pH, nitrogen source and other certain factors within the cytoplasm of host macrophages^17,18^.

### Cytokine response to *T. marneffei* infected THP-1

**Figure 9:**
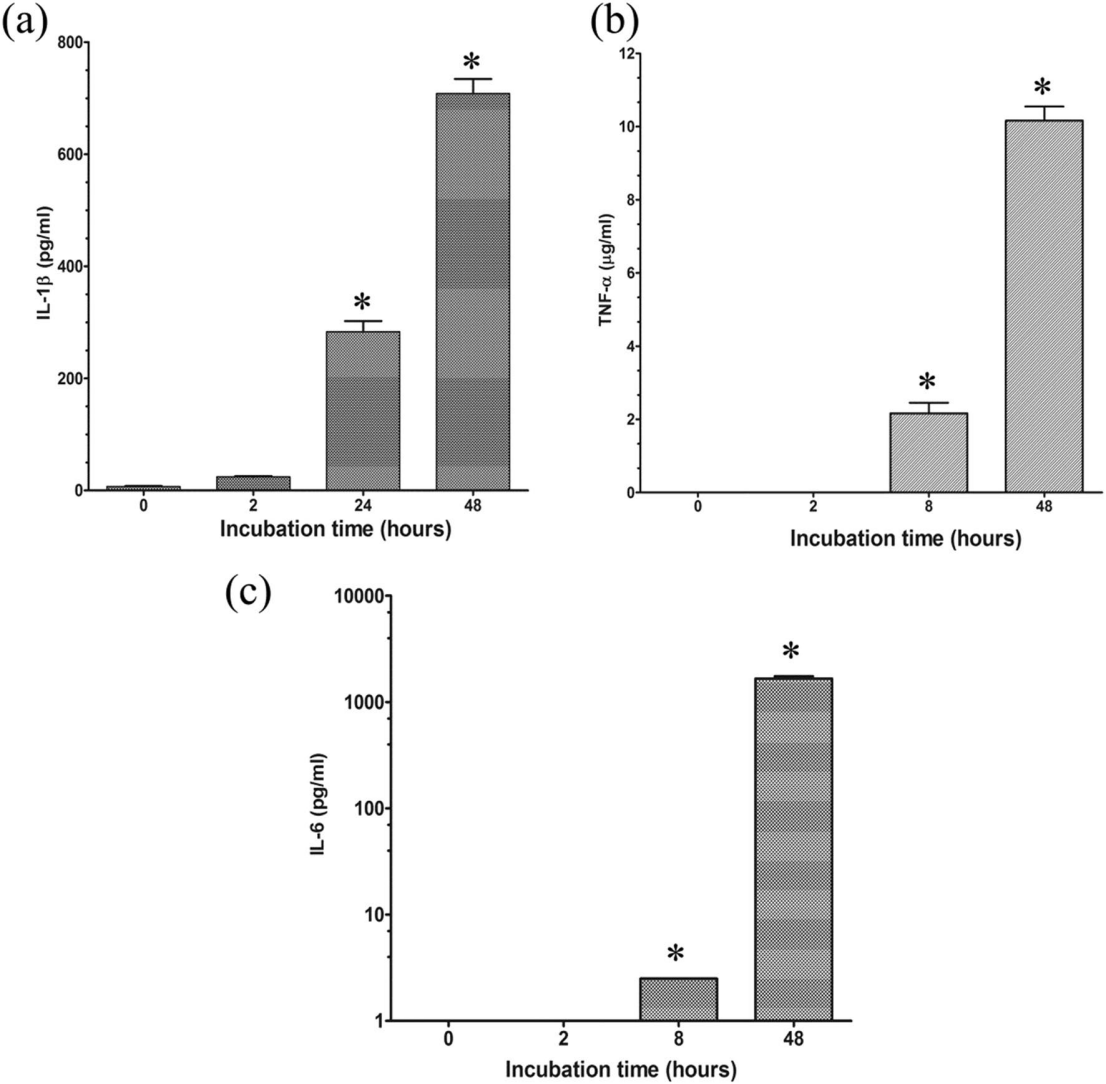
Pro-inflammatory cytokine release from THP-1 macrophages co-cultured with conidia from *T. marneffei* at different time points. (**a**) Interleukin 1β (IL-1β), (**b**) tumor necrosis factor alpha (TNF-α) and (**c**) interleukin 6 (IL-6). Measurements of TNF-α, IL-6, and IL-1β levels were achieved using supernatants pooled from three sets of experiments and expressed as mean ± SD. An asterisk (*) indicates a significant difference (*p* < *0.05*) from supernatants at 8, 24 and 48 h compared with 0 h.

To investigate the role of host macrophage cytokine responses during phase transition and replication in *T.* *marneffei*, the levels of pro-inflammatory cytokines TNF-α, IL-6, and IL-1β secretion were examined after incubation with THP-1 cells at different time points. It was observed that during the early stage of 2 h after internalization, the cytokine levels were not detected. After longer incubation times (8–48 h), the concentrations of TNF-α, IL-6 and IL-1β secreted from infected THP-1 were significantly increased, consistent with the progress of the conidia to the yeast conversion and replication (Fig. 9).

## Discussion

It has long been thought that reversible morphogenesis from an environmental mold to a pathogenic yeast is a requisite for the pathogenicity of systemic dimorphic fungi, including *T.* *marneffei*. This remarkable event is stimulated by exposure to host factors, especially host body temperature, and leads to genetic programming needed for adaptation to the host environment, including genes for promoting survivability and virulence^4,5^. Conidia inhaled into the host lung are internalized by alveolar macrophages. Within the cytoplasm of macrophages, the conidia of *T.* *marneffei* form unicellular yeast cells, which then divide by fission^1^.

In our studies, MAb 4D1 was specifically reactive against yeast phase antigen of *T.* *marneffei*. This immunoreactivity was detected in both cytoplasmic antigens and cell wall associated antigen recovered from *T.* *marneffei* undergoing thermally induced conidia or mycelial to yeast transition. Moreover, MAb 4D1 recognized the mannoprotein antigens in the yeast phase, which was not detected in the mold phase, confirming the alteration of cell wall properties during phase conversion in *T.* *marneffei*. Normally, mannoprotein is synthesized in the cytoplasm and then translocated and accumulated on the cell wall of the fungus through the yeast secretory pathway^19,20^. The evidence from the IFA clearly demonstrated that target protein of MAb 4D1 could be associated with the cell surface of *T.* *marneffei* yeast cell. Thus, the reconstitution of cell surface molecules in yeast cells of *T.* *marneffei* is essential for the survival, and may assist fungal adaptation to host conditions, and to escape host elimination, in a manner similarly exhibited by other dimorphic fungi^21^.

The biochemical characterization of TM CYA clearly showed that the antigenic target recognized by MAb 4D1 was highly glycosylated. Antibody cross–reactivity arising from the presence of glycans containing epitopes in the pathogenic fungal antigen has been frequently described^22–25^. However, digestion with PNGase F in our experiment altered the recognition pattern of TM CYA from the native broad high molecular mass smear to neo-diffused immunoreactive bands with lower molecular weight of approximately 30 kDa. In contrast, the immunoreactivity against TM CYA was completely abolished after treatment with proteinase K. These observations imply that the target epitope of MAb 4D1 is a peptide and may not be the glycan components. This could be a basic reason for the high degree of specificity exhibited by MAb 4D1. Moreover, O-linked deglycosylation with O–glycosidase did not alter the recognition pattern of MAb 4D1^7^. This result suggests that the antigenic target of MAb 4D1 was an N (asparagine)-linked glycoprotein.

The presence of N-linked glycans in a fungal glycoprotein is invariably associated with the presence of a mannan group^26^; and this would suggest the identity of this antigen as a mannoprotein. The GNA lectin binding studies clearly revealed that mannose is the main glycosylation constituent of TM CYA. On the other hand, Rafferty demonstrated that some lectins do not recognize the common glycans in TM CYA including a sialic acid attached carbohydrate (when investigated by *Sambucus nigra* lectin and *Maackia* *amurensis* lectin) or O-linked glycans (when investigated by Peanut agglutinin)^7,27^.

MAb 4D1 was generated in the pre-proteomes and recombinant protein technology era^7,10^ and the antigenic target recognized by this clone is unknown. Purification of MAb 4D1 target proteins from numerous contaminating proteins in TM CYA have been attempted by liquid isoelectric focusing (IEF). However, purification results have showed that MAb 4D1 is reactive against the peptide which has several differing isoelectric points (pIs) ranging from 3.2 to 9.6 (unpublished data). As a result, the micro-heterogeneous property of MAb 4D1 target proteins is suspected. According to the deglycosylation and IEF purification results, it is likely that the micro-heterogeneity is due to different degrees of glycosylation in the MAb4D1 target protein. Such micro-heterogeneity phenomena have been observed in yeast glycoproteins including *Paracoccidioides* *brasiliensis* and *Saccharomyces* *cerevisiae*^28,29^. Further studies (e.g., affinity pull-down assay or immunoprecipitation) are necessary to investigate and to identify the MAb 4D1 target proteins and specific epitopes using proteomic analysis protocols.

Several antigenic glycoproteins from cell wall associated and secreted forms of *T.* *marneffei* are readily isolated from its crude protein extracts. For example, concanavalin A recognize mannose moieties in both mycelial and yeast phase unidentified antigens of *T.* *marneffei*^30^. Furthermore, the cell wall mannoprotein Mp1p is differentially expressed in both the mycelial and yeast phases of *T.* *marneffei*^31^. Although many antigenic proteins of *T.* *marneffei* were reported with mannose glycosylation, this novel antigenic mannoprotein target of TM CYA recognized by MAb 4D1 is distinct from those previously described. When compared to Mp1p, which has a molecular mass of 58 and 90 kDa and is expressed in both mycelial and yeast phases, the MAb 4D1 target proteins have a broad high molecular mass smear pattern with a MW of approximately 50–150 kDa and expressed in only the yeast phase.

The thermal dimorphism of *T.* *marneffei* plays an important role in the establishment of infection. Alteration in the expression of cytoplasmic or cell wall associated proteins have been observed during the phase transition of dimorphic fungi for example, heat shock proteins^32–34^ and some immunogenic mannoproteins such as Mp1p and Mplp6^31,35^. Moreover, during the morphological transition from conidia to yeast, modification of glycan cell wall was demonstrated. In *Blastomyces* and *Paracoccidioides*, the percentages of immunogenic *β*-(1, 3)-glucan in the hyphal cell wall (5%) were less than that in the yeast cell wall (40%)^36,37^. The *T.* *marneffei* novel yeast phase-specific proteins were expressed during the phase transition from conidia to yeast cell. Thus, the yeast phase-specific proteins are likely to play important roles in immunogenicity, virulence and pathogenicity. We believe that our investigation focusing on MAb 4D1 will be an important prototypic model in the study on the relationship between *T.* *marneffei* phase transition and its virulence; and the MAb 4D1 target protein could be used as a rapid yeast phase-specific marker (expressed within 12 h after internalization) inside the THP-1 human macrophage.

The present study demonstrates that *T.* *marneffei* is capable of intracellular survival, phase transition, and replication within macrophages. These events partly direct fungal pathogenesis and can modify the innate immune response in early phases of infection. We found that TNF-α is significantly increased after longer incubation in THP-1, cells suggesting that the yeast forms of *T.* *marneffei* could elicit higher production of this pro-inflammatory cytokine. In addition, both IL-1 β and IL-6 were significantly elevated after shifting from conidia to yeast cells. We noted that the host phagocytes could mount an immune response to halt the progression of infection. In this regard, Dong et al. recently demonstrated that the appropriate proinflammatory cytokines production in AIDS-associated talaromycosis plays a beneficial role in protective immunity and the survivability of patients^38^.

In addition to thermally induced phase transition, several other stimuli could influence morphogenesis including oxidative stress, changes in CO_2_ tension, steroid hormones, acidic pH, nitrogen source, and other factors within the cytoplasm of macrophages^17,18^. To establish infection after entering the lungs, fungal conidia encounter professional phagocytes, including neutrophils and macrophages. There they must also combat reactive oxygen and nitrogen species such as nitric oxide, superoxide anion, hydrogen peroxide and hydroxyl radicals. Macrophages rapidly generate reactive oxygen and nitrogen species, which impair the proteins, lipids, and nucleic acids of invading microorganisms, eventually eliminating them^39^. For example, TNF-α production increases the capacity of macrophages to combat fungal pathogens since it enhances IFN-γ production and induces reactive oxygen and nitrogen species production that kill fungi or suppress their growth^40^.

In summary, the pathogenicity of *T.* *marneffei* seems to be vitally based on their ability to undergo a phase transition and display multiple morphotypes with different surface properties. Our present study demonstrates that MAb 4D1 can be applied as a biomolecular tool for understanding the phase transition of *T.* *marneffei,* and provides strong evidence for this fungal shift from an environmental saprophyte to a pathogenic fungus. Future studies will hopefully potentially identify additional protective effects and further the understanding of MAb 4D1’s therapeutic potential and its use in possible passive immunization.

## Materials and methods

### Fungal isolates

*T.* *marneffei* ATCC 200051 was used for all experiments in both the mycelial form and yeast form, previously isolated from a bone marrow sample of a patient infected with HIV at Maharaj Nakorn Chiang Mai Hospital, Chiang Mai, Thailand. The *T.* *marneffei* isolate was maintained by monthly subculture onto Potato Dextrose Agar (PDA; Difco). *T.* *marneffei* was grown on PDA for 5 days at 25 °C. The conidia were removed from the surface of the PDA by washing the surface growth with 5 ml of sterile PBS and gentle scraping with a cotton swab. The resulting culture suspension was then filtered through sterile glass wool and centrifuged at 5000 g for 15 min followed by three washes with sterile PBS. In addition, other fungal isolates were obtained and cultivated according to the directions from the American Type Culture Collection (ATCC) or from Department of Medical Services, Ministry of Public Health, Bangkok, Thailand. The fungal strains used in all experiment were summarized in Table 2.

**Table 2:** Fungal isolates.

Fungal species (abbreviation)	Isolate number
*Talaromyces marneffei* (TM)	ATCC 200051^δ^
*Sporothrix schenckii* (SS)	52-S1^†^
*Histoplasma capsulatum* (HC)	53-H1^†^
*Candida albicans* (CA)	ATCC 900028^δ^
*Candida krusei* (CK)	CI
*Candida glabrata* (CG)	CI
*Cryptococcus neoformans* (CN)	H99^δ^
*Aspergillus fumigatus* (AF)	55-A1^†^
*Penicillium citrinum* (PC)	MMC59P12‐1^Ϩ^
*Pythium insidiosum* (PI)	MMC44P21-1^Ϩ^
*Pseudallescheria boydii* (PB)	MMC60S21‐1^Ϩ^
*Trichosporon* *sp.*	CI
*Geotrichum sp.*	CI

^δ^Isolate from the American Type Culture Collection, Rockville, MD, USA.

^†^Isolates from the Institute of Dermatology, Department of Medical Services, Ministry of Public Health, Bangkok, Thailand.

^Ϩ^Isolate from culture collection in Mycology Unit, Department of Microbiology, Faculty of Medicine, Chiang Mai University, Chiang Mai, Thailand.

CI: Clinical isolates from blood samples of infected patients.

### *T. marneffei* cytoplasmic yeast antigen (TM CYA) extraction

In order to investigate the expression of a yeast specific antigen in *T.* *marneffei*, the fungus, at concentration 1 × 10^6^ cells/ml, was inoculated in several 250-ml flasks containing 50 ml of brain heart infusion broth (BHI; Difco) with shaking at 150 rpm at 37 °C over a period of time. An individual culture was then periodically harvested at every 6 h after initially harvesting the 24 h culture flask. The fungal cells were then harvested by centrifugation after treatment with 0.02 g of thimerosal (Sigma) per liter at room temperature for 24 h. The preparation of *T.* *marneffei* cytoplasmic yeast antigen (TM CYA) was carried out as previously described^41^. The protein concentration was determined by the dye binding method^42^ and protein bands were analyzed by 10% SDS-PAGE followed by staining with Coomassie Blue (InstantBlue, Expedeon). The cytoplasmic mold or yeast antigens of the other fungi were prepared following the same procedures.

### Purification of monoclonal antibody 4D1 (MAb4D1) and specificity testing

The hybridoma cell line clone 4D1 was maintained in serum free medium (Gibco), and purified by HiTrap column protein G affinity chromatography (GE Healthcare) according to methods previously described^9^. Immunoreactivity determination of MAb 4D1 was carried out using indirect ELISA with various fungal antigens and slide culture based indirect immunofluorescence assay (IFA)^8,43^.

### Antigenic determinants characterization and *Galanthus nivalis* agglutinin (GNA) binding assay

TM CYA deglycosylation reactions were investigated using GlycoProfile II commercially available kits (Sigma) containing recombinant peptide N-glycosydase F (PNGase F). All reactions were carried out as per the manufacturer’s instructions. Briefly, 10 µg of TM CYA were mixed with denaturing buffer (2% octyl β-D- glucopyranoside, 100 mM 2-mercaptoethanol) and incubated at 95 °C for 10 min. Once the mixed solution had cooled to room temperature, reaction buffer (20 mM ammonium bicarbonate) and PNGase F (2.5 enzyme units) were added and the mixture reaction was incubated at 37 °C for 90 min or 180 min, respectively. After that, mixture was then investigated by immunoblotting by using MAb 4D1 as previously described^8,9^.

For proteinase K digestion, TM CYA (10 µg) were dissolved in reaction buffer (1.0 M sorbitol, 0.1 M sodium citrate, pH 5.5) supplemented with 5 µg/ml of recombinant proteinase K (Roche). The mixture solution was then incubated at 37 °C for 30 min or 60 min, respectively. The remained immunoreactivity of MAb 4D1 was investigated with immunoblotting as mentioned above.

The nature of carbohydrate components of the glycoprotein present in TM CYA was determined using horseradish peroxidase conjugated *Galanthus* *nivalis* agglutinin (HRP-GNA) or snowdrop lectin. HRP-GNA was incorporated into the immunoblotting format of SDS-PAGE separated proteins. Briefly, 10 µg of TM CYA were subjected to SDS-PAGE gel and transferred onto nitrocellulose membrane (Hybond extra, Amersham). The membranes were blocked with PBS containing 5% (w/v) skim milk (Sigma). After washing with phosphate buffered saline containing 0.05% Tween 20 (PBST), membranes were incubated with 1:5,000 HRP-GNA (EY Laboratory Inc. USA) in PBST containing 2.5% (w/v) skim milk for 60 min. The membranes were washed three times with PBST, and the bound conjugate was developed visualization by incubation in ready-to-use TMB-substrate solution (Invitrogen, Ca, USA). The reactions were stopped by submersion the membrane in distilled water.

### LC–MS analysis

The identification of 72 kDa TM CYA antigen recognized by HRP-GNA were carried out as described previously^44^.

#### In gel digestion

Protein bands were excised, the gel plugs were dehydrated with 100% acetonitrile (ACN), reduced with 10 mM DTT in 10 mM ammonium bicarbonate at room temperature for 60 min and alkylated at room temperature for 60 min in the dark in the presence of 100 mM iodoacetamide (IAA) in 10 mM ammonium bicarbonate. After alkylation, the gel pieces were dehydrated twice with 100% ACN for 5 min. To perform in-gel digestion of proteins, 10 µl of trypsin solution (10 ng/µl trypsin in 50% ACN/10 mM ammonium bicarbonate) was added to the gels followed by incubation at room temperature for 20 min, and then 20 µl of 30% ACN was added to keep the gels immersed throughout digestion. The gels were incubated at 37 °C for a few hours or overnight. To extract peptide digestion products, 30 µl of 50% ACN in 0.1% formic acid (FA) was added into the gels, and then the gels were incubated at room temperature for 10 min in a shaker. Peptides extracted were collected and pooled together in the new tube. The pool extracted peptides were dried by vacuum centrifuge and kept at − 80 °C for further mass spectrometric analysis.

#### Liquid chromatography-Tandem mass spectrometry (LC/MS–MS)

The tryptic peptide samples were prepared for injection into an Ultimate3000 Nano/Capillary LC System (Thermo Scientific, UK) coupled to a Hybrid quadrupole Q-T of impact II (Bruker Daltonics) equipped with a Nano-captive spray ion source. Briefly, one microlitre of peptide digests were enriched on a µ-Precolumn 300 µm i.d. X 5 mm C18 Pepmap 100, 5 µm, 100 A (Thermo Scientific, UK), separated on a 75 μm I.D. × 15 cm and packed with Acclaim PepMap RSLC C18, 2 μm, 100 Å, nanoViper (Thermo Scientific, UK). The C18 column was enclosed in a thermostatted column oven set to 60 °C. Solvent A and B containing 0.1% formic acid in water and 0.1% formic acid in 80% acetonitrile, respectively were supplied on the analytical column. A gradient of 5–55% solvent B was used to elute the peptides at a constant flow rate of 0.30 μl/min for 30 min. Electrospray ionization was carried out at 1.6 kV using the Captive Spray. Nitrogen was used as a drying gas (flow rate about 50 l/h). Collision-induced-dissociation (CID) product ion mass spectra were obtained using nitrogen gas as the collision gas. Mass spectra (MS) and MS/MS spectra were obtained in the positive-ion mode at 2 Hz over the range (m/z) 150–2200. The collision energy was adjusted to 10 eV as a function of the *m*/*z* value.

#### Bioinformatics and data analysis

The MS/MS data from LC–MS analysis were submitted for a database search using the Mascot software^45^ (Matrix Science, London, UK). The data was searched against the NCBI database for protein identification. Database interrogation was; taxonomy (*Talaromyces marneffei* or *Penicillium* *marneffei*); enzyme (trypsin); variable modifications (carbamidomethyl, oxidation of methionine residues); mass values (monoisotopic); protein mass (unrestricted); peptide mass tolerance (1.2 Da); fragment mass tolerance (± 0.6 Da), peptide charge state (1+ , 2+ and 3+) and max missed cleavages.

### Antigenic glycoprotein expression profiling during phase transition using MAb 4D1 in an indirect ELISA assay

In order to investigate the effect of culture temperature on protein expression profiles in *T.* *marneffei*, the fungus was cultured in BHI broth at both temperature, 25 °C and 37 °C. The conidia, at concentration 1 × 10^6^, were inoculated in BHI broth at 37 °C or 25 °C on an orbital shaking incubator at 150 rpm and harvested at 6 h intervals between 24 to 60 h. The cultures were killed by the addition of 0.02% (w/v) thimerosal solution, followed by incubation at room temperature for 24 h. Killed *T.* *marneffei* cultures were harvested by centrifugation for preparing TM CYA as previously described.

Maxisorp 96-well microtiter plates were coated with 0.5 μg/ml of TM CYA which were prepared at different time points and diluted in carbonate buffer (pH 9.4). The plates were incubated overnight at 4 °C then washed three times with PBST and were blocked by incubation with 200 μl of 1% bovine serum albumin (BSA) in PBST for 60 min at 37 °C. After three additional washes with PBST, 2.5 µg/ml of MAb 4D1 were added to each well and incubated at 37 °C for 60 min. After being washed as described above, the plates were incubated at 37 °C for 60 min with 100 µl of HRP conjugated goat anti-mouse IgG (Jackson, West Grove, Pa.) at 1:10,000 in blocking solution. The plates were then washed twice with PBST and once with PBS only. One hundred µl of ready-to-use TMB-substrate solution (Invitrogen, Ca, USA) was added and the ELISA-plate was incubated for 15 min in the dark. The reaction was stopped by the addition of 50 µl of 2.0 N H_2_SO_4._ The optical densities (OD) at 450 nm were measured on an ELISA reader (Shimadzu model UV-2401PC, Kyoto, Japan). The assay was repeated three times for at least two independent assays, and the results are expressed as mean OD for each determination.

### Quantification of *T. marneffei* conidia uptake by human monocytic cell line THP-1 cells by flow cytometry

The human monocytic cell line THP-1 (ATCC TIB-202) was cultured in RPMI 1640 medium (Gibco, USA) containing 10% (v/v) heat-inactivated FBS (Gibco). For the induction of cell differentiation, cells (2 × 10^6^ per ml) were seeded into 24-well culture plates (Costar, Corning, NY) in 1 ml of RPMI 1640 medium with 10% (v/v) FBS and 50 ng/ml of phorbol myristate acetate (PMA) (Sigma) for 48 h. After incubation, adherent cells were washed with RPMI-1640 three times. THP-1 cells in RPMI 1640 without PMA were used as control cells. The conidia suspended in RPMI 1640 medium with 10% (v/v) FBS were added to the wells containing a monolayer of THP-1. Then, THP-1 cells were allowed to ingest *T.* *marneffei* conidia at MOI of 5 for 2 h. After incubation, non-internalized conidia were eliminated and killed with 50 μg/ml of nystatin as described^46^. THP-1 cells were then supplemented with fresh media for an additional different time points of 0, 12 to 60 h. THP-1 were removed from the wells with 0.25% trypsin–EDTA (Gibco) for 5 min at 37 °C and washed twice to remove trypsin–EDTA, and then fixed by adding 4% paraformaldehyde in cold PBS (Sigma) and lysed with 1% Triton-X 100 in PBS for 15 min. Fungal cells were then washed with PBS 5 times and stained with MAb 4D1, at concentration 0.1 mg/ml, for 2 h at 37 °C. After washing 5 times, Alexaflor 488 conjugated goat anti-mouse IgG (Invitrogen), at dilution 1:500 was added and incubated for 2 h at 37 °C. After 3 washing with PBS, *T.* *marneffei* cells were counted by Flow cytometer and the percentages of positive cells with MAb 4D1 were calculated. Flow cytometry was performed on a BD FACSAria with BD FACSDiva application software version 5.0.2 (BD Biosciences, Franklin Lakes, NJ) paired with FlowJo Version 6.3.2 analysis software (Tree Star Inc., Ashland, OR). A total of 10,000 cell events were analyzed at wavelength 495–520 nm. Unstained control conidia and yeast cells were analyzed for relative cell size and subtracted the autofluorescence background. The experiments were performed in triplicates and analyzed using standard t-test (http://www.graphpad.com/quickcalcs/ttest1.cfm Format = SD).

### The percentage of *T. marneffei* yeast cells cultured in vitro (37 °C) at different time points as quantitated by flow cytometry

This method was modified from that described by Batista et al.^47^, *T. marneffei* conidia, at concentration 2 × 10^6^ were inoculated in 1% proteose peptone (Difco) with shaking at 150 rpm at 37 °C over a period of time. An individual culture was then periodically harvested at every 12 or 24 h of time intervals during 24 to 144 h. The fungal cells were then harvested by centrifugation after adding 0.02 g of thimerosal per liter at room temperature for 24 h. The cells were washed 5 times with PBS, and then stained with MAb 4D1 as mentioned above. The cells were quantified by flow cytometry as described above, and then analyzed using standard t-test (https://www.graphpad.com/quickcalcs/ttest1/?Format=SD).

### Determination of conidia to yeast transition in *T. marneffei* in THP-1 cells by using immunofluorescence assay

To characterize yeast cell transition inside the macrophage cell line, THP-1 monocytes were seeded at 10^6^ cells/well in 6 well plate containing coverslip and were differentiated as described above. Undifferentiated monocytes were removed by washing with PBS. *T. marneffei* conidia were labeled with 0.1 mg/ml fluorescein isothiocyanate (FITC, Sigma-Aldrich, St. Louis, USA) in 0.1 M carbonate buffer (pH 9.0) at 4 °C for overnight with shaking^48^. Labeled conidia were then extensively washed three times with PBS containing 0.1% (v/v) Tween 20 and then counted under fluorescence microscope. The labeled conidia were added to THP-1 cells at a multiplicity of infection (MOI) of 5 and allowed to ingest *T. marneffei* conidia for 2 h as described above. After removing non-internalized conidia and then treated with nystatin, THP-1 cells were supplemented with fresh media for an additional different time points 0, 8, 12, 24, and 36 h. At the end of each incubation period, cells were fixed for 10 min with 4% paraformaldehyde in cold PBS, and permeabilized with 0.2% Triton-X 100 in PBS, for 10 min. The cells were then washed with PBS 5 times and incubated 2 h at 37 °C with the MAb 4D1 at concentration 0.1 mg/ml. The coverslips were washed three times with 2% PBS–BSA and then incubated for 1 h with the secondary antibody. Alexaflor 555 conjugated goat anti-mouse IgG (Invitrogen) was used at 1:500 dilution. Cells were then washed three times with PBS. Following another round of washings, cells were labeled with 4′,6-diamidino-2-phenylindole or DAPI (0.5 μg/ml; Sigma) in PBS for 5 min before a single final wash in PBS. Images were acquired using a Nikon DS Fi1.

### Enumeration of fungal viability and induction of cytokines in *T. marneffei* infected THP-1

To investigate the survival of *T.* *marneffei* inside THP-1, the experiment was carried out as described above. After that, infected THP-1 was lysed by the adding 1.0% of triton X-100 followed by serially diluted of the released fungal cell and plating onto the PDA and incubating at 25 °C for 72 h. The colony forming unit (CFU) of *T.* *marneffei* from cell lysates after 2 h of phagocytosis was used to establish a CFU control for comparison with the CFUs at subsequent time intervals.

The culture supernatants of *T.* *marneffei* infected THP-1 of each time point (0, 2, 8, 24 and 48 h) were collected and kept at − 80 °C until the cytokine assays were performed. TNF-α, IL-6 and IL-1β were measured by commercial double antibody sandwich ELISA kits (BioLegend, San Diego, CA), according to the manufacturer’s instructions. The experiments were performed in triplicates and then analyzed as previously described^49^.

## Supplementary information

Supplementary Figures.
