# Supplementary material for: Characterization of a novel yeast phase-specific antigen expressed during in vitro thermal phase transition of Talaromyces marneffei
Source: Sci Rep. 2020 Dec 3;10:21169. doi: 10.1038/s41598-020-78178-5 (PMC7713699; doi:10.1038/s41598-020-78178-5)
Supplement: Supplementary file 1 — Supplementary Figures. [file 41598_2020_78178_MOESM1_ESM.docx]

**Supplementary data**

**Characterization of a novel yeast phase-specific antigen expressed during**

***in vitro* thermal phase transition of *Talaromyces marneffei***

# Authors: Kritsada Pruksaphon, Mc Millan Nicol Ching, Joshua D. Nosanchuk, Anna Kaltsas, Kavi Ratanabanangkoon, Sittiruk Roytrakul, Luis R. Martinez, and Sirida Youngchim

**
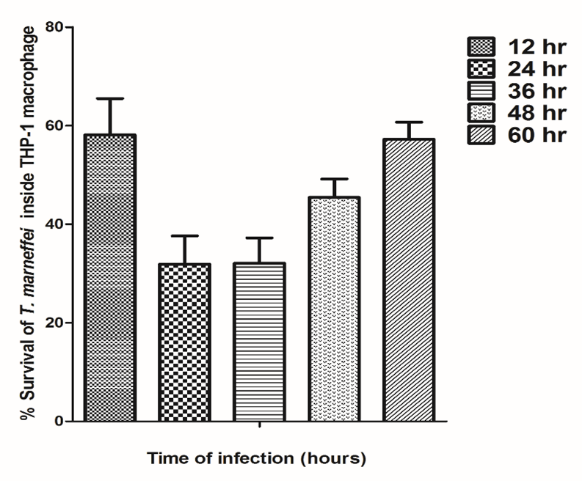
**

**Supplementary Figure S1.** The percentage of *T. marneffei* survivability inside THP-1 macrophages at 12, 24, 36, 48, and 60 hours respectively confirmed the ability of fungus to survive and replicate inside the macrophages. The infected THP-1 cell lysates after 2 hours of phagocytosis were used to establish a CFU control, normalized with the CFUs at subsequent time intervals. The bars indicate mean ± SD of triplicated experiment.

**
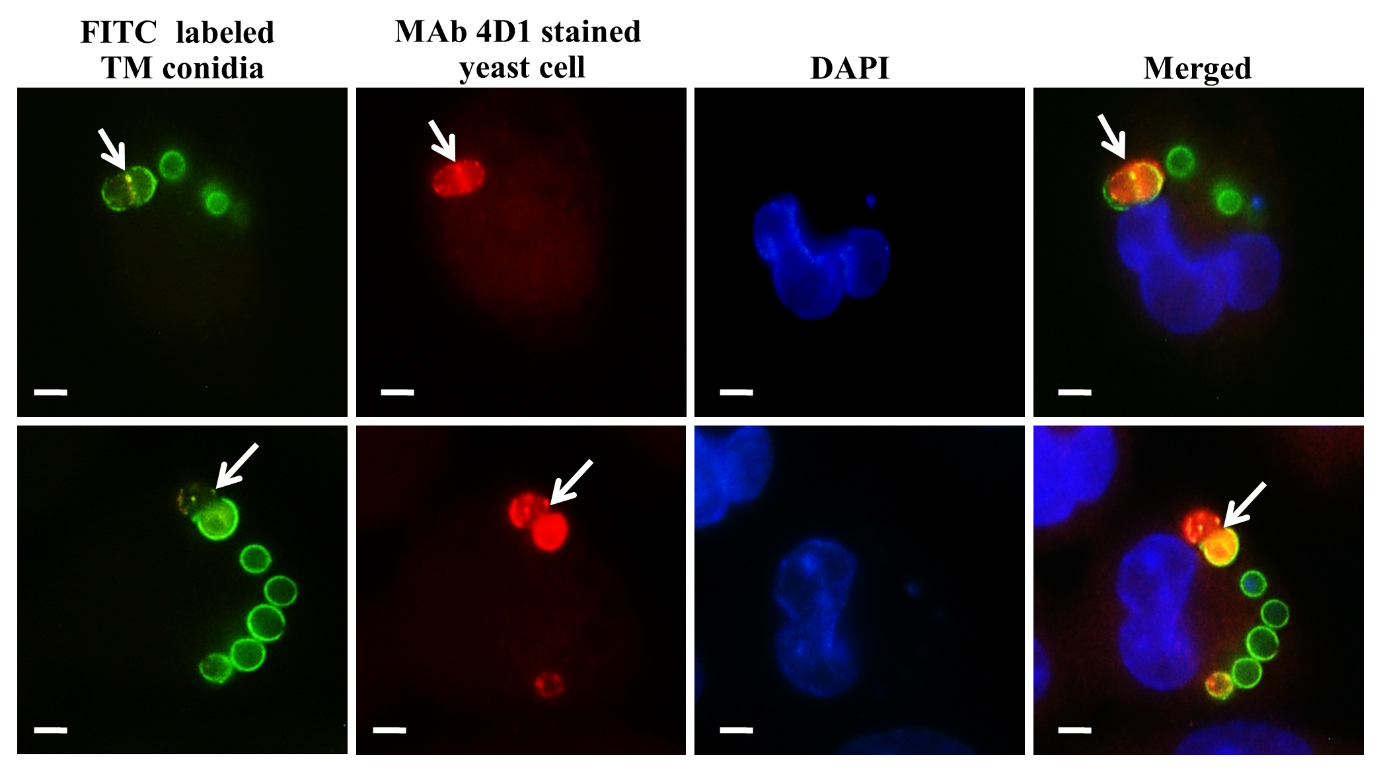
**

**Supplementary Figure S2.** The overlapping signals between the green of FITC labeled conidia and the red of MAb4D1 which gives the co-localized signal as a yellow at 12 hours after internalization. *T. marneffei* yeast cells were labeled with MAb 4D1 and Alexaflor 555 conjugated goat anti-mouse IgG antibody. From left to right: fluorescence image showing the green channel (FITC labeled conidia); fluorescence image of the red channel (MAb 4D1 positive yeast cells); THP-1 nuclei were stained with DAPI (blue) a merged channel showing the overlapping of triple images. Bars, 5 µm
